# Pediatric genetic counselor use and perception of various clinic models

**DOI:** 10.1002/jgc4.70028

**Published:** 2025-04-30

**Authors:** Emily Franciskato, Elly Brokamp, Elizabeth Jasper, Christine Munroe, Jacquelyn Britton, Laura Duncan

**Affiliations:** ^1^ Master of Genetic Counseling Program Vanderbilt University Nashville Tennessee USA; ^2^ Department of Obstetrics and Gynecology Vanderbilt University Medical Center (VUMC) Nashville Tennessee USA; ^3^ Department of Pediatrics VUMC Nashville Tennessee USA; ^4^ Department of Biomedical Informatics VUMC Nashville Tennessee USA; ^5^ Primary Care Precision Medicine Clinic, Department of Family Medicine University of Pittsburgh Pittsburgh Pennsylvania USA; ^6^ Department of Genetic Medicine Johns Hopkins University Baltimore Maryland USA; ^7^ Present address: Vanderbilt Genetics Institute Nashville Tennessee USA; ^8^ Present address: Mayo Clinic Center for Individualized Medicine; ^9^ Present address: Vanderbilt University Medical Center Department of Obstetrics and Gynecology; ^10^ Present address: Vanderbilt University Department of Biomedical Informatics

**Keywords:** genetic counseling services, pediatrics, practice models, service delivery models

## Abstract

Pediatric genetic counseling consistently has the longest referral to appointment time among all genetic counseling specialties. Pediatric genetic counselors (GCs) are utilizing non‐traditional clinic models (NTM) in their practices. This study identified the clinic models utilized by GCs and describes GCs' perceptions regarding clinic model use and implementation. Pediatric GCs were recruited via the National Society of Genetic Counselors student research email. Descriptive statistics were used to describe respondent demographics, clinic model types utilized, average monthly patient volume, and genetic counselor perceptions of their clinic model, including barriers, helpful resources, and perceived improvements with NTM implementation. Of the 78 respondents, 80.8% of pediatric GCs were working in a NTM role at least some of the time. The average monthly patient volume was higher for GCs exclusively in NTM and combination clinic roles versus GCs solely in traditional model (TM) roles. The genetic counseling‐only clinic was the NTM utilized most among NTM and combination GCs. A higher proportion of GCs working with NTMs agreed they were satisfied with their job, felt supported in the clinic, were at the top of their scope, and provided better patient care than TM GCs. Barriers exist to NTM implementation, and the process was not standardized across clinics. This study provides preliminary evidence showing NTM use can increase pediatric GC patient volume while increasing job satisfaction.

**What is known about this topic**

Pediatric GCs are practicing within different types of clinic models.

**What this paper adds to the topic**

Identifies the types of clinic models pediatric GCs are utilizing, GCs preferences for clinic models used, and strategies for different clinical model implementation.

## 1 INTRODUCTION

Since launching the Service Delivery Model Task Force in 2009, the National Society of Genetic Counselors (NSGC) has conducted broad overviews of service delivery models (SDMs) used in genetic counseling in the United States from 2010 to 2020 (Boothe et al., 2021; Greenberg et al., 2020). Alternative SDMs can increase access to genetic counseling by increasing the number of patient visit slots without decreasing patient satisfaction (Greenberg et al., 2020). Based on these studies, the genetic counseling field should continue to assess increasing access to GC services using alternative SDMs.

Genetic counseling had a projected job growth rate of 29%–72% from 2014 to 2024. According to the US Labor Bureau, a growth rate of 16% is predicted through 2032 (Bureau of Labor Statistics, 2023; Hoskovec et al., 2018; Martiniano et al., 2016). Despite the job growth in the profession, pediatric GC wait times have increased through the years. Only 6% of the 50 pediatric GC respondents to the 2017 Professional Status Survey reported the ability to accommodate patients in less than a week (Greenberg et al., 2020). Pediatric GCs report the longest wait time to the next available appointment option in both 2010 and 2017 compared to non‐pediatric specialties. The majority (54%) of pediatric GCs stated their wait time was >2 months (Greenberg et al., 2020). This contrasts with prenatal GCs who reported the majority (55%) of their patients are scheduled within 1 week and cancer GCs who reported the majority (57%) of their patients are scheduled within 2 weeks (Greenberg et al., 2020). It is hypothesized that access to a board‐certified medical geneticist may contribute to appointment wait times for pediatric GCs (Boothe et al., 2021; Cohen et al., 2013). According to the American Board of Genetics and Genomics, the number of board‐certified medical geneticists has increased by less than one thousand individuals since 2009 (Maiese et al., 2019). Pediatric geneticists also had the longest appointment wait times among geneticist specialties. Most patients waited more than 3 months for appointments (Maiese et al., 2019).

There are reports of pediatric GCs currently taking the initiative to employ non‐traditional models (NTMs) in their clinics (Kubendran et al., 2017). One NTM optimized consultation time with the medical geneticist by having patients see a local GC and pediatrician in person. The pediatrician and GC would triage patients to a remote medical geneticist if indicated. Before implementing the NTM, patients waited an average of 6 months for a genetics evaluation due to medical geneticist availability. When this NTM was in use, the pediatrician and GC initiated evaluation within an average time of 6 weeks. The pediatrician and GC team identified 34 patients, 13% of study individuals, who did not need a geneticist evaluation. This eliminated unnecessary geneticist visits, thus freeing up slots for appropriately referred patients. The statistical significance of wait times for genetics prior to NTM usage was not assessed in this study due to the referral triage system in use. Triage would extend the wait time for less urgent patients and allow for syndromic and other emergency patients to be seen more quickly (Kubendran et al., 2017).

A 2020 survey of all NSGC members described the necessity of alternative SDMs and barriers to alternative SDM implementation. A majority (54.4%) of the 517 respondents declared their current SDM(s) were inadequate for addressing clinical needs (Boothe et al., 2021). The majority (64.8%) of respondents were exploring the implementation of a new SDM to increase the number of patients and access in remote areas, decrease wait times, improve the quality of care, and create clinical efficiency despite low numbers of GCs, genetic counseling assistants (GCAs), administrative assistants, and medical geneticists (Boothe et al., 2021). Some common barriers reported to SDM implementation included billing and licensure obstacles, understaffing in genetic counseling clinics, time constraints, and funding concerns (Boothe et al., 2021).

There is immense interest in expanding the utilization of NTMs in genetic counseling to keep pace with the growing need for GCs as genetic technology evolves (Boothe et al., 2021; Cloutier et al., 2017; Cohen et al., 2013; Greenberg et al., 2020; Trepanier & Allain, 2014). To our knowledge, a quantitative or qualitative comprehensive review of clinic models utilized in pediatric genetics has not been completed. This study aimed to quantitatively characterize pediatric genetic counseling clinic models in use as well as GCs' perspectives of these models. This includes barriers, successes, possible improvements, and helpful resources for NTM implementation. For the sake of this study, a clinic model is any SDM or clinic flow that could improve patient care by direct‐to‐patient or direct‐to‐provider interaction. Open‐ended responses were included to identify any pertinent topics not directly addressed in this new survey and provide leads for future research on this topic. For this study, the traditional pediatric genetic counseling model (TM) is defined as a clinic flow where the GC and medical geneticist see a patient in a single visit. Additionally, a non‐traditional pediatric genetic counseling clinic model (NTM) is defined as any clinic flow differing from the traditional model.

## 2 MATERIALS AND METHODS

A confidential online survey of current pediatric GCs was constructed to gather information surrounding pediatric genetic counseling clinic models. This survey contained both closed and open‐ended responses.

### 2.1 Recruitment

Individuals were recruited via the NSGC student research email blast sent to all members of NSGC. An additional email was sent a week later via the same listserv. One month later researchers gathered email addresses of pediatric GCs open to student contact using the “pediatrics” filter on the NSGC “Find a Genetic Counselor” function (*N* = 107). Identified GCs worked at an institution not currently represented by survey respondents after the first two email blasts. Individual emails containing the same blast message were sent to these GCs to increase response numbers.

### 2.2 Eligibility

Eligible participants self‐identified as English‐speaking pediatric GCs. GCs could work in clinics at academic medical centers, hospitals, and private practices. Participants could operate in any specialty, including general pediatric genetics, pediatric oncology, specialty pediatric clinics, or others not listed. The GCs could operate in any clinical role: seeing patients full time, seeing patients part time, research, and leadership. Individuals who did not self‐identify as pediatric GCs were excluded from the study.

### 2.3 Survey instrumentation

A 72‐question survey was developed by researchers with dichotomous choice, multiple choice, check all that apply, and open response questions. Branching logic ensured participants received questions based on their specific clinic model experience. A copy of this survey is available in Appendix S1. Survey questions focused on pediatric models in use, GC demographics, and personal average monthly patient volume. Closed and open‐ended questions identified other professionals present in the clinic, interprofessional interactions with the GC, and interprofessional interactions with patients. GCs' perceptions of their current clinic model were assessed. These constructs included job satisfaction, barriers to NTM implementation, and successes with NTM implementation. Beliefs regarding GC colleagues' clinic model perceptions were also measured. Individuals had the opportunity to list their institution to allow for analysis between different respondents from the same institution.

The developed survey was piloted by members of the research committee and revised for readability. Responses were collected and managed using REDCap electronic data capture tools hosted at Vanderbilt University (Harris et al., 2009, 2019). The final survey question asked GCs to voluntarily share their email for a randomized Amazon gift card drawing. Voluntary responses were stored separately from other responses to ensure anonymity.

### 2.4 Data analysis

Researchers utilized R for descriptive data analysis and figure creation (R Core Team, 2021). Basic descriptive statistics were gathered for dichotomous choice, multiple choice, and select all that apply questions (Wickham et al., 2019). The mean, median, range, and standard deviation were calculated for personal average monthly patient volume. Given the quantitative aims defined for this study, a complete thematic analysis of responses to open‐ended questions was not completed for this study. Authors elected to include open‐ended responses regarding different clinic model aspects given the novelty of this survey and topic. Some responses will be included in a “How to Implement a New Clinic Model” guide to demonstrate the possible utility of a future thematic analysis as it relates to pediatric clinic models. Seven quotes were edited to remove potentially identifiable information such as city for the respondent or start dates. These details were replaced with a “…” or “[information for understanding]” to increase anonymity. All open‐ended responses were included in Table S1 for viewing.

## 3 RESULTS

There was a total of 97 responses received correlating to a response rate of 12% assuming all pediatric GCs who completed the 2020 PSS received one of the email blasts (“NSGC Professional Status Survey: Executive Summary,” 2020). Of these responses, 19 were eliminated due to incomplete surveys. A total of 78 responses met inclusion criteria for this study, and 15 of these responses were received after individually addressed emails were sent. The majority of respondents had 1–4 years of experience as a GC (42/78; 53.8%), followed by 5–9 years (17/78; 21.8%), less than a year and 10–14 years (7/78 9.0%), and finally 20 plus years (4/78; 5.2%). The total years of pediatric GC experience corresponded similarly with the majority reporting 1–4 years as a pediatric GC (45/78; 57.7%), followed by 5–9 years (19/78; 24.4%), less than a year (9/78; 11.5%), 10–14 years (4/78; 5.1%), and 20 plus years (1/78; 1.3%). A breakdown of GC demographics by clinic model is available in Table S2. Respondents report utilizing TMs, NTMs, and a combination of the two. More respondents practiced solely with the TM (15/78; 19.2%), while 12.8% practiced with only NTMs (10/78). Most individuals used a combination of models in their clinic (53/78; 67.9%; Table S2).

### 3.1 Types of NTMs in use

Given the responses of those practicing solely in NTM and those using a combination of models showed similar clinic model utilization patterns, results for these groups were combined in this section. The NTM utilized most was genetic counseling only clinic (47/63; 74.6%), with over three quarters of respondents billing for time spent using this model (36/47; 76.6%). Another common NTM was a genetic counselor working with a non‐genetics provider (37/63; 58.7%), with the majority of GCs billing for time spent (21/37; 56.8%). The non‐genetics clinics mentioned in this study include neurology, cardiology, immunology, plastic surgery, gastroenterology, nephrology, urology, and neonatology. Respondents reported using all [ten] types of NTMs listed on the survey, and eight additional NTMs were described, highlighting the variety of NTMs available. Table 1 lists all reported NTMs and the billing statuses associated with them.

**TABLE 1:** A breakdown of the NTMs in use and billing information for those models from 63 NTM users.

Non‐traditional models used	*N* (%)	Yes bill (*N*/%)	No bill (*N*/%)
GC only clinic	47 (74.6%)	36 (76.6%)	11 (23.4%)
GC working with non‐genetics providers	37 (58.7%)	21 (56.8%)	16 (43.2%)
GC triages referrals	35 (55.6%)^a^	1 (2.9%)	33 (97.1%)
GC on‐call	21 (33.3%)^a^	2 (10.0%)	18 (90.0%)
NP working with GC in TM	17 (27.0%)	14 (82.4%)	3 (17.6%)
Medical geneticist only visits	17 (27.0%)	13 (76.5%)	4 (23.5%)
MD sees the patient, and sees GC as needed	17 (27.0%)	10 (58.8%)	7 (41.2%)
GC to non‐genetics provider consult	15 (23.8%)	4 (26.7%)	11 (73.3%)
Utilization of GCAs	15 (23.8%)	0 (0.0%)	15 (100%)
GC completes phone intake	10 (15.9%)	0 (0.0%)	10 (100%)
NP working the medical geneticist in TM	4 (6.3%)	3 (75.0%)	1 (25.0%)
GC with PCP	4 (6.3%)^a^	0 (0.0%)	3 (100%)
Genetic test review process outside of clinic	4 (6.3%)	0 (0.0%)	4 (100%)
Utilization of pre‐recorded videos	2 (3.2%)	0 (0.0%)	2 (100%)
Other	8 (12.7%)^a^	4 (57.1%)	3 (42.9%)

^a^ Indicates that one GC in this group did not answer whether they bill for their time spent in this model.

The NTMs identified were implemented in the years 2000–2021, with over 96% beginning after 2011. Many individuals stated their NTM is not formally reassessed on a specific timeline (25/63; 39.7%). A smaller portion of individuals had a formal assessment annually, monthly, or quarterly (18/63; 28.6%). Multiple individuals stated their clinics reassess their NTM as needed.

### 3.2 Average monthly patient volume

**FIGURE 1:**
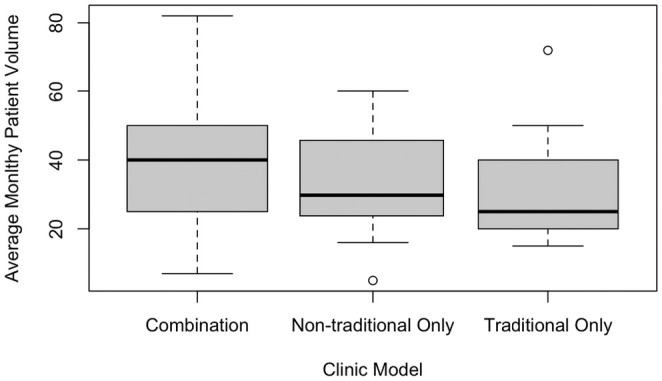
Reported GC personal average monthly patient volume.

GCs using some form of NTM saw a higher average of patients per month than those using only TMs. TM GCs reported a personal average monthly patient volume of 30.93 ± 16.18 patients. They saw a median of 25 patients per month (Figure 1). GCs practicing in NTMs alone reported seeing 33.44 ± 15.69 patients on average per month. They saw a median of 30 patients per month. For those using a combination of the TM and NTMs, the total average monthly patient volume was 40.02 ± 17.03 patients, with a median of 40 patients per month (Figure 1). For combination GCs an average of 51.38% of the GCs time was spent using a NTM in clinic. Written responses hypothesized their increased patient volume was due to decreased wait time. One GC shared, “GC only clinic…has a shorter wait time. Medical geneticist clinic wait time is 4–6 months. My GC only clinic has availability within 1–2 weeks.”

### 3.3 Perceived barriers and difficulties with NTM implementation

TM GCs identified several barriers to implementing a NTM at their clinic. The barrier most identified by TM GCs was billing complications (13/15; 86.67%). Lack of available administrative support (12/15; 80%) and complications encountered with new workflows (11/15; 73.3%) were also possible barriers for NTM implementation. Other less frequently possible barriers were budgeting and interprofessional team hesitation (8/15; 53.3% respectively), student involvement (4/15; 26.7%), and difficulties with role delegation (2/15; 13.3%). Some GCs reported a lack of confidence managing referrals, screenings, and follow‐up testing independently (2/15; 13.3%), and some GCs specifically reported low confidence working without medical geneticist (MD) support as a barrier (2/15;13.3%). One GC stated they perceived hospital system regulations as another barrier not explicitly stated in this survey. A summary of these perceived barriers compared to actual encountered difficulties by NTM GCs is available in Table 2.

**TABLE 2:** Summary of perceived NTM implementation barriers, NTM implementation difficulties, and helpful NTM implementation resources.

Perceived barriers to NTM implementation by TM participants	*N* = 15
Billing	13 (86.7%)
Lack of administrative support	12 (80.0%)
Logistics with setting up the new workflow	11 (73.3%)
Budgeting	8 (53.3%)
Interprofessional team hesitation or medical geneticist resistance	8 (53.3%)
Time	6 (40.0%)
Student involvement	4 (26.7%)
Difficulty delegating roles	2 (13.3%)
Handling no‐shows	2 (13.3%)
Lack of confidence managing patient (referrals, screening, follow‐up testing)	2 (13.3%)
Lack of confidence without support of medical geneticist	2 (13.3%)
Other	1 (6.7%)
Hospital system regulations	1
**Experienced barriers with NTM implementation by all NTM participants**	***N* = 63**
Logistics with setting up the new workflow	34 (54.0%)
Interprofessional team hesitation or medical geneticist resistance	25 (39.7%)
Billing	23 (36.5%)
Time	23 (36.5%)
Lack of administrative support	22 (34.9%)
Referral backlog	16 (25.4%)
Budgeting	11 (17.5%)
Difficulty delegating roles	11 (17.5%)
Handling no‐shows	8 (12.7%)
Lack of confidence without medical geneticist support	6 (9.5%)
Student involvement	5 (7.9%)
Lack of confidence managing patient referrals, screening, and follow‐up testing	4 (6.4%)
Other	10 (15.9%)
None	3
NTM already in place at employment start	4
Changing scheduling protocol/view in electronic medical record to fill NTM clinics	1
Building up referral base	1
Licensure	1

Helpful resources for NTM implementation	*N* = 63
Weekly clinic meetings	24 (38.1%)
Hiring GCAs	23 (36.5%)
Clear mentoring process for new GCs	14 (22.2%)
New features on the electronic medical record	4 (6.4%)
Other	13 (20.6%)
Clinic personnel buy‐in	4
Collaborations with other clinics	2
Scheduler education on appropriate GC referrals	2
NSGC special interest groups	1
Monthly division meeting discussions	1
Research grants to help fund NTM implementation	1
Unknown	1
Not involved with NTM implementation	1

NTM GCs most often encountered difficulties setting up new workflows when implementing a NTM (34/63; 53.97%). Other common difficulties included interprofessional team hesitation or medical geneticist resistance (25/63; 39.7%), billing (23/63; 36.5%), time (23/63; 36.5%), lack of administrative support (22/63; 34.9%), referral backlogs (16/63; 25.4%), budgeting (11/63; 17.5%), difficulty delegating roles (11/63; 17.5%), handling no‐shows (8/63; 12.7%), lack of confidence without MD support (6/63; 9.5%) and a lack of confidence managing patients (4/63; 6.4%), and student involvement (5/63; 7.9%). A third of GCs who used a NTM identified billing as a difficulty when implementing their clinic's NTM (23/63; 36.51%). Some participants stated this model was already in use when they began working, so they could not identify barriers for their institution. In addition, some participants affirmed they did not encounter any difficulties when implementing a NTM. A comparison of these perceived barriers and difficulties is available in Table 2.

**FIGURE 2:**
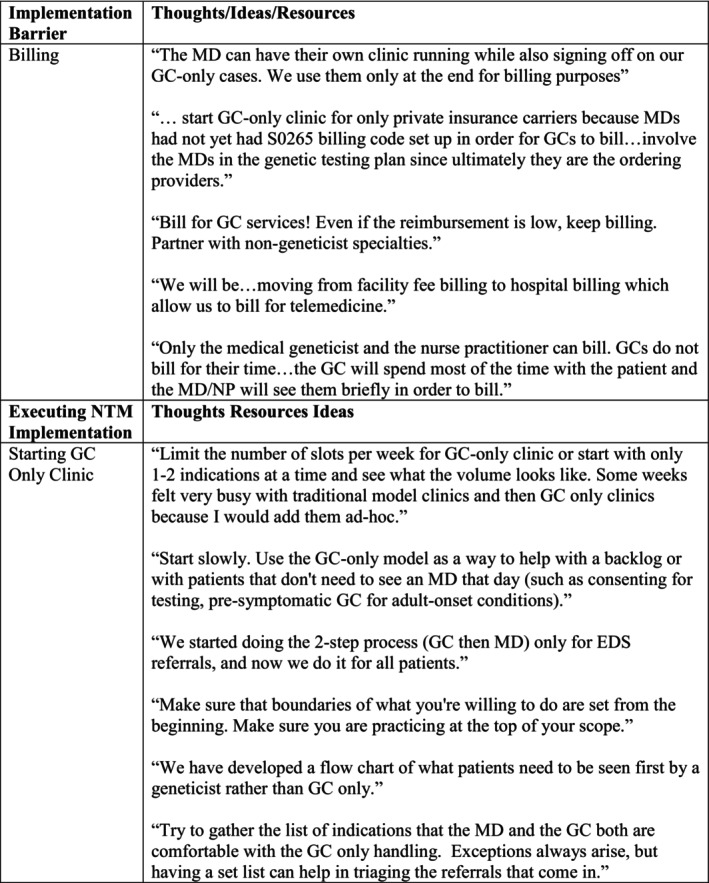
Possible suggested NTM implementation guide layout.

Open‐ended responses from NTM and combination GCs shared encouragement and ideas to combat perceived barriers anticipated by TM GCs, as well as identified implementation difficulties for NTM and combination GCs. A summary of some of these thoughts is included in Figure 2 to demonstrate the possible utility of a clinic model implementation guide.

### 3.4 Helpful resources for implementation

Most TM GCs stated hiring genetic counseling assistants (GCAs) would be a helpful resource for NTM implementation (10/15; 66.7%), but GCs who used NTMs had a smaller majority who felt hiring GCAs was helpful for implementation (23/63; 36.51%). TM GCs stated new features in the EMR would be a helpful resource (6/15; 40.0%). In contrast, a smaller percentage of NTM GCs stated new features in the EMR were helpful for implementation (4/63; 6.35%). The resource most NTM individuals found helpful with implementation was weekly clinic meetings (24/63; 38.1%), which were reflected by the TM expectations of helpful resources. Both groups stated that having clear mentoring processes for new GCs is a perceived and actual helpful resource for implementation. Other helpful resources identified by NTM GCs not listed on the survey included helping schedulers discern which patients needed a medical geneticist versus a GC appointment and overall collaboration with other professionals both at an individual's own clinic and beyond. One participant was not involved in their NTM implementation and was unable to identify helpful resources. Helpful resource responses by NTM users are summarized in Table 2.

### 3.5 Perceived improvements due to NTM implementation

NTM GCs witnessed improvements with use of a NTM in clinic. The most common improvement was decreased wait time for patients (44/63; 69.8%). Over half of NTM GCs also reported increased job satisfaction (35/63; 55.6%) and increased appointment flexibility (36/63; 57.1%). Close to half of NTM GCs saw increases in provider confidence in the GC (31/63; 49.2%). Other improvements included increased funding for more GCs, GCAs, medical geneticists, and other personnel (13/63; 20.63%). A small number of GCs were unsure about any noted improvements since NTM implementation (6/63; 9.5%), and an even smaller number reported no perceived improvements (2/63; 3.2%). There were multiple other perceived improvements with the NTMs included as free responses such as new billing abilities, more time for GCs to support patients benefiting most from GC involvement thus decreasing burnout risks, increased access to care across all clinics, more efficient testing processes, and decreased referral to testing time. Many GCs shared written sentiments regarding autonomy; one GC notes, “…this model has also greatly increased GC satisfaction by giving us greater autonomy in the pediatric setting.”

### 3.6 Genetic counselor perceptions of roles in clinic models

**FIGURE 3:**
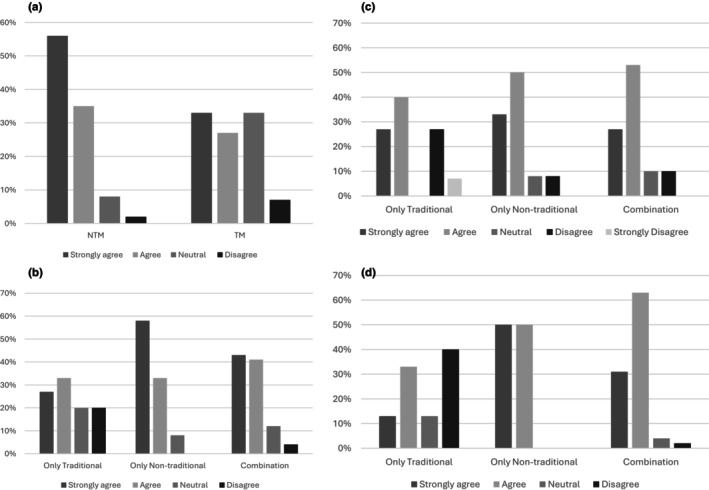
Breakdown of genetic counselors' perceptions of respective clinic models (Put in formation of 4). (a) Agreement with “A new clinic model allows me to provide better patient care.” Asked to NTM users and agreement with “A new clinic model would allow me to provide better patient care” asked to TM users. (b) Agreement with “I feel supported by other professionals in clinic.” (c) Agreement with “I am working at my top scope of practice.” (d) Agreement with “I am satisfied with my job.”

Over half of the TM GCs agreed or strongly agreed that a NTM would allow for better patient care (9/15; 60.0%). The overwhelming majority of NTM users agreed or strongly agreed that their NTM allowed them to provide better patient care (57/63; 90/5%; Figure 3a). Just over half of TM GCs agreed or strongly agreed about feeling supported in clinic (9/15; 60.0%), and a fifth of TM GCs disagreed about feeling supported in clinic by other clinic professionals (3/15; 20%). In contrast, the vast majority of NTM and combination model GCs agreed or strongly agreed they felt supported by others in clinic (54/63; 85.7%; Figure 3b). A majority of TM GCs agreed or strongly agreed they were operating at the top of their scope of practice (10/15; 66.7%). A higher majority of NTM and combination model GCs felt they were operating at the top of their scope of practice (51/63; 81.0%; Figure 3c). Just over half of TM GCs agreed or strongly agreed they were satisfied with their job (7/15; 46.7%). Over a third of GCs working exclusively in the TM disagreed with the statement, “I am satisfied with my job” (6/15; 40.0%; Figure 3d). For GCs utilizing a NTM or combination models a vast majority agreed or strongly agreed they were satisfied (60/63; 95.3%; Figure 3d).

Most TM GCs would be likely or very likely to try a NTM in clinic if opportunities presented (13/15; 86.7%). In contrast, most TM GCs perceived their institution and MDs would be unlikely or very unlikely to implement a NTM in the future (9/15; 60.0% and 8/15: 53.5% respectively). A smaller majority of TM GCs felt the geneticists would harbor neutral feelings regarding a NTM (4/15; 26.7%), and the smallest fraction of GCs perceived the MDs to be open to a NTM (3/15; 20.0%).

### 3.7 Clinic model preferences

Of the 78 individuals, one GC has practiced exclusively in the TM, and six GCs have practiced exclusively in a NTM throughout their careers. For the 71 individuals who had experience with both TM and NTM, preference between TM and NTM was measured. Most of the GCs surveyed preferred or strongly preferred using a NTM in their clinic (41/71; 57.7%). A smaller number of GCs did not have a preference between the two clinic model types (22/71; 30.9%). Finally, the smallest percentage preferred or strongly preferred use of the TM over use of a NTM (8/71; 11.3%). Of the GCs currently working in the TM, one GC's clinic was planning to implement a NTM within the next 5 years, eight GCs were unsure, and five GCs stated their clinic was not moving toward NTM implementation. GCs written responses reflected how preferences could depend on indications seen in their NTM clinic. One NTM GC shared, “I like the autonomy … and being the genetics expert in a department outside of General Genetics.” While another NTM GC thought, “GC only visits tend to be adults who are more worried…with fam history or kids with variants on previous genetic testing, which is not as interesting to me.” A visual graph of GC clinic model preferences is available in Figure S1.

## 4 DISCUSSION

The current pediatric genetic counseling landscape is not meeting the demand for services the field faces (Boothe et al., 2021; Cohen et al., 2013). This study identified different clinic models utilized across pediatric genetic counseling, including NTMs. Patient volume increased for most GCs utilizing NTMs. Implementation of NTMs had obstacles. GCs provided ideas to combat these issues, and some GCs have witnessed improvements in clinic, such as increased autonomy and overall job satisfaction. NTMs have the potential to positively impact the pediatric genetic counseling landscape.

Addressing the demand for GC services by increasing patient volume and decreasing wait time from referral to appointment was a proposed goal of non‐traditional SDM implementation in previous studies (Boothe et al., 2021; Greenberg et al., 2020). GCs using NTMs in this study were seeing more patients per month on average than GCs using TMs alone. This trend was examined in one GC‐only clinic at an academic medical center. Their GC‐only visits were scheduled an average of 132 days earlier than visits involving the MDs (King et al., 2022). These findings suggest NTMs may assist with long pediatric genetic counseling wait times, thus increasing access to GC services.

Common barriers to non‐traditional SDM implementation in genetic counseling for all specialties include billing, lack of support including staffing, administrative, physician, institutional, and funding, as well as time (Boothe et al., 2021). Similar barriers to NTM implementation and use were identified by pediatric GCs. Like previous studies, billing problems with alternative models were a common theme (Boothe et al., 2021; Greenberg et al., 2020). A small majority of GCs utilizing NTMs identified billing as a barrier when they were implementing their NTM (36.5%), which may imply this barrier is not as prevalent as perceived. Some study participants believe the COVID‐19 pandemic eliminated the barrier of billing for the telehealth services. NTM GCs that use telehealth services can now bill when it was previously restricted by most institutions before the pandemic (Boothe et al., 2021; Greenberg et al., 2020). Many clinics in this study changed their workflow to increase the number of patient slots without sacrificing revenue due to billing restrictions. These models highlight the impact billing status has on access to genetics care and provide further evidence of the benefits of GCs being recognized as billing providers. Several individuals stated gathering billing data in different types of pediatric clinic models would be helpful for the future.

A lack of staffing and administrative support continues to be a major perceived barrier to implementing NTMs in clinics. Genetic counseling assistants (GCAs) have been a common target for eliminating some of the administrative burdens faced by GCs (Pirzadeh‐Miller et al., 2017). Only 25% of NTM participants reported the use of a GCA in their clinics. GCAs could be an underutilized resource when considering NTM implementation. Further research may help understand the lack of GCAs in this population and identify novel roles for GCAs in pediatric genetics clinics moving forward.

Survey respondents curated a list of helpful resources for NTM implementation. The first key resource in the beginning stages of NTM implementation is collaborator buy‐in. To achieve this, participants suggested identifying clinic leadership who support the use of NTMs and proposing ideas with that collaborator. Locating an administrative collaborator who was interested in clinic operations and services also helped streamline processes for some respondents. Some GCs reported gaining administrative support approval was easiest when the referral list was long and wait times were increasing. Multiple participants concluded implementation did not happen quickly, so having collaborators who advocated with GCs and convinced other providers was key.

Some GCs shared their successes when first implementing their NTM. Respondents suggested creating a list of indications that are referrals for medical geneticists versus GCs. This idea was executed in practice and was successful for a previously reported NP/GC clinic (Stewart & Svihovec, 2022). In this clinic, patients referred to genetics for hypermobile Ehlers–Danlos syndrome or neurofibromatosis type I evaluations were triaged to the NP/GC clinic and seen 6–9 months earlier than in the MD/GC clinic (Stewart & Svihovec, 2022). Starting small gave the clinic time to adjust to the change over time, address unforeseen difficulties, and combat certain barriers to billing that could present (Stewart & Svihovec, 2022). Addressing the most pressing needs of the clinic allowed for a greater opportunity to install small pieces of NTMs over time. Study data implied NTM implementation is not a standardized process across institutions, making implementation at new facilities difficult. Providing targeted guides on implementation could alleviate some stressors faced by providers implementing NTMs and potentially lead to a greater success with NTMs overall. Using data from the qualitative responses, the authors created an example of a NTM implementation guide in Figure 2.

In this study, GCs shared the positive impacts that NTM implementation had on their jobs and clinics. Many NTM GCs reported seeing patients independently. This independence could be leading to higher job satisfaction, as supported by the number of NTM GCs satisfied or very satisfied with their jobs in this study. Increases in collaboration also showed positive impacts in NTM clinics. GCs in NTMs worked collaboratively with a diverse set of providers. Specialty clinics could be potential areas to increase the presence of pediatric genetic counseling services. Previously, inpatient genetic counselors identified several benefits to working on interprofessional teams with non‐genetics providers (Clark et al., 2021). These benefits included increased collaboration for more consistent care, increased referrals from non‐genetics services to the genetics clinic, and opportunities to educate non‐genetics providers about the value of genetics in medical care (Clark et al., 2021). It is reasonable to hypothesize that these same benefits seen in the inpatient space could be contributing to the increases found in job satisfaction for those in pediatric genetic counseling. Overall, the autonomy provided by working in GC‐only clinics, as well as the opportunities for multidisciplinary collaboration and support in other NTMs, have shown the possibility to decrease wait time for patients, as well as increased GC satisfaction in their NTM clinics.

Most GCs utilizing a NTM reported a preference for their NTM. It is possible that this preference contributed to the higher levels of job satisfaction reported for NTM users, but further research is needed to confirm such hypotheses. It is important to note not every individual favored their clinic's NTM over the TM. One GC reported indications for their GC‐only clinic were less interesting than when they were working with the MD in the TM. GCs also reported troubles with delegating time and roles when deciding the number of patients to see weekly in the NTM. For some patients, seeing a medical geneticist and GC at the same time could be most beneficial for their care, so it is important to recognize these situations and understand how the clinic will address this. Findings in this study suggested the presence of numerous objective, subjective, and institutional factors impacted model use and success in pediatric clinics.

### 4.1 Study limitations

Survey responses were ascertained through NSGC listservs and the NSGC “Find a genetic counselor” feature. Responses may not reflect the perceptions of all pediatric GCs. The response rate did not allow for stratified comparisons between clinic model groups due to the ratio of NTM to TM users. This uneven ratio could suggest that most clinics are utilizing a NTM of some kind. Another limitation is the presence of recruitment bias: pediatric GCs with a desire to change their clinic model or already practicing in a NTM may have been more likely to fill out this survey about clinic models. An emerging practice in pediatric genetics is inpatient genetic counseling (Clark et al., 2021). This study did not explore how clinic models incorporate inpatient counseling into their practices. Additionally, the small sample size of this study limited the ability to investigate associations between clinic models used and GC perceptions. Given the limited responses and aims of this study, full thematic analysis of all open‐ended responses was not completed, so at this time it is not known if these responses clearly delineate specific themes regarding pediatric clinic models.

### 4.2 Future directions

This and past studies highlight the desire for NTM and alternative SDM use in genetic counseling. Pediatric GCs in this study reported personal benefits of increased GC autonomy. Some GCs have also seen an increase in patient volume. Lack of billing and administrative support is a barrier to implementing NTM in clinics. A larger survey with current and past pediatric GCs could increase sample sizes and allow for linear and multivariate regression analysis to establish relationships between clinic model type, GC satisfaction, and average monthly patient volume. Qualitative interviews with pediatric GCs who combated perceived barriers to implementing NTMs in their clinics could give a detailed description of helpful resources. This could help create standardized resources for NTM implementation and use. Several individuals desired patient satisfaction data for clinics using a NTM. Additional comparisons of patient satisfaction data before and after NTM usage could assist with overcoming institutional barriers to NTM implementation. Assessing main factors contributing to reported job satisfaction may identify specific improvements to strive for when implementing a NTM. Additional research regarding clinic models in pediatric genetic counseling could be a focal point of innovation and collaboration among pediatric GCs to increase access to genetic services and advocate for professional growth.

## 5 CONCLUSIONS

Pediatric GCs were working in the TM, NTMs, and a combination of both model types. The type of model and process of NTM implementation was not standardized across clinics. Most TM individuals were interested in implementing new clinic models at their institutions despite the barriers in place. When pediatric GCs in this study utilized a NTM, they generally had greater job satisfaction and feelings of support from others in clinic and felt they were operating at the top of their scope when compared to the TM group in this study. NTMs allowed for greater autonomy in the clinic for individuals as well as increased patient volume. Pediatric GCs may find these considerations helpful in leading the way for the implementation of a NTM in their workplace.

Further research has the capability to assist in NTM implementation tool creation and increase the opportunity for pediatric genetic counseling expansion and access to pediatric genetics services.

## AUTHOR CONTRIBUTIONS

Authors Emily Franciskato and Laura Duncan confirm that they had full access to all the data in the study and take responsibility for the integrity of the data and the accuracy of the data analysis. All authors gave final approval of this version to be published and agree to be accountable for all aspects of the work in ensuring that questions related to the accuracy or integrity of any part of the work are appropriately investigated and resolved.

## ETHICS STATEMENT

Emily, Laura, Elly, Elizabeth, Christine, and Jacquelyn all declare that they have no conflict of interest. This study was approved by and conducted according to the ethical standards of the Vanderbilt University Institutional Review Board. All applicable international, national, and/or institutional guidelines were followed. This study was approved by the IRB after expedited review and was granted an informed consent waiver (#211136).

## Supporting information


Figure S1

Table S1

Table S2

Appendix S1

## Data Availability

Data available upon request to the authors.
