# Supplementary material for: Pediatric genetic counselor use and perception of various clinic models
Source: J Genet Couns. 2025 Apr 30;34(3):e70028. doi: 10.1002/jgc4.70028 (PMC12043028; doi:10.1002/jgc4.70028)
Supplement: Supplementary file 1 — Figure S1 [file JGC4-34-0-s004.docx]

Supplementary Figure 1: GC preference of clinic model if they worked in both TM and NTM
