# Supplementary material for: Pediatric genetic counselor use and perception of various clinic models
Source: J Genet Couns. 2025 Apr 30;34(3):e70028. doi: 10.1002/jgc4.70028 (PMC12043028; doi:10.1002/jgc4.70028)
Supplement: Supplementary file 2 — Table S1 [file JGC4-34-0-s001.docx]

Supplementary Table 1: Demographics of 78 survey respondents. Regions listed are defined by National Society of Genetic Counselors.

| **Total Years Experience** | **Total** | **TM** | **NTM** | **Combination** |
| --- | --- | --- | --- | --- |
| <1 | 7 | 2 | 0 | 5 |
| 1 to 4 | 42 | 10 | 2 | 30 |
| 5 to 9 | 17 | 3 | 2 | 12 |
| 10 to 14 | 7 | 0 | 3 | 4 |
| 20 to 24 | 1 | 0 | 1 | 0 |
| 25+ | 3 | 0 | 2 | 1 |
| **Total Years Peds Experience** | **Total** | **TM** | **NTM** | **Combination** |
| <1 | 9 | 3 | 0 | 6 |
| 1 to 4 | 45 | 9 | 5 | 31 |
| 5 to 9 | 19 | 3 | 3 | 13 |
| 10 to 14 | 4 | 0 | 2 | 2 |
| 20 to 24 | 0 | 0 | 0 | 0 |
| 25+ | 1 | 0 | 0 | 1 |
| **Region** | **Total** | **TM** | **NTM** | **Combination** |
| Region 1 | 2 | 1 | 0 | 1 |
| Region 2 | 15 | 5 | 1 | 9 |
| Region 3 | 8 | 2 | 1 | 5 |
| Region 4 | 35 | 6 | 4 | 25 |
| Region 5 | 9 | 1 | 2 | 6 |
| Region 6 | 9 | 0 | 2 | 7 |
