# Supplementary material for: Pediatric genetic counselor use and perception of various clinic models
Source: J Genet Couns. 2025 Apr 30;34(3):e70028. doi: 10.1002/jgc4.70028 (PMC12043028; doi:10.1002/jgc4.70028)
Supplement: Supplementary file 4 — Appendix S1 [file JGC4-34-0-s003.docx]

1. Do you identify as a pediatric genetic counselor? This can include seeing patients full-time or part-time, leadership, research, or other roles in a pediatric genetic counseling setting.
   1. Yes
   2. No
2. What is your race/ethnicity?
   1. American Indian or Alaskan Native
   2. Asian
   3. Asian Indian
   4. Black or African American
   5. Middle Eastern
   6. Native Hawaiian or Other Pacific Islander
   7. White
   8. Other
   9. Prefer not to respond
3. How many total years of experience do you have as a genetic counselor?
   1. <1 year
   2. 1-4 years
   3. 5-9 years
   4. 10-14 years
   5. 15-19 years
   6. 20-24 years
   7. 25+ years
4. How many total years of experience do you have as a pediatric genetic counselor?
   1. <1 year
   2. 1-4 years
   3. 5-9 years
   4. 10-14 years
   5. 15-19 years
   6. 20-24 years
   7. 25+ years
5. What region as defined by NSGC is your clinic in?
   1. Region 1 (CT, MA, ME, NH, RI, VT, CN)
   2. Region 2 (DC, DE, MD, NJ, NY, PA, VA, WV, PR, VI, Quebec)
   3. Region 3 (AL, FL, GA, KY, LA, MS, NC, SC, TN)
   4. Region 4 (AR, IA, IL, IN, KS, MI, MN, MO, ND, NE, OH, OK, SD, WI, Ontario)
   5. Region 5 (AZ, CO, MT, NM, TX, UT, WY, Alberta, Manitoba, Sask.)
   6. Region 6 (AK, CA, HI, ID, NV, OR, WA, British Columbia)
6. For purposes of data analysis what is the name of the institution you practice at? We will not publish the names of institution. We will be looking at differences between individuals in the same clinic if more than one individual from your clinic takes this survey.
7. What pediatric genetic counseling clinic model do you currently utilize at your institution? Researchers for this study define traditional pediatric genetic counseling model as: genetic counselor has face to face conversation with patient and will elicit medical and family history and provide genetic counseling as needed, report to medical geneticist, and genetic counselor and medical geneticist have face to face conversation regarding care plan. Some examples of non-traditional clinic models include genetic counseling only clinic, genetic counselor working with a nurse practitioner, genetic counselor seeing patients after the medical geneticist as needed, genetic counselor to non-genetics provider consult, etc.
   1. Only traditional
   2. Only non-traditional
   3. Combination traditional of non-traditional

**Only using the traditional model:**

1. What professionals are present in your clinic that interact with the same patients you do? (select all that apply)
   1. Medical geneticist
   2. Nurse practitioner
   3. Genetic counseling assistant
   4. Administrative team
   5. Primary care physicians
   6. Other (please specify)
2. What is your average patient volume (unique patients for which you participated in care with the medical geneticist) in the traditional model per month in your clinic? (Free response) *put as an integer
3. What perceived barriers are there to implementing a new clinic model at your clinic?
   1. Lack of administrative support
   2. Logistics with setting up the new workflow
   3. Budgeting
   4. Referral backlog
   5. Lack of confidence without medical geneticist support
   6. Lack of confidence managing patient (referrals, screening, follow-up testing)
   7. Interprofessional team hesitation or medical geneticist resistance
   8. Difficulty delegating roles
   9. Billing
   10. Student involvement
   11. Handling no-shows
   12. Time
   13. Other (free response)
4. What other resources could your institution provide to help implement non-traditional models?
   1. New features on the EMR
   2. Hiring GCA’s
   3. Clear mentoring process for new GCs
   4. Clinic meetings weekly
   5. Other (please specify)
5. If you have practiced with both the traditional model and a non-traditional clinic model, which clinic model do you prefer between the non-traditional and traditional?
   1. Strongly prefer traditional model
   2. Prefer traditional model
   3. Neutral
   4. Prefer non-traditional model
   5. Strongly prefer non-traditional model
   6. I have only practiced with a traditional model.
6. How much do you agree with this sentence: I am satisfied with my job.
   1. Strongly agree
   2. Agree
   3. Neutral
   4. Disagree
   5. Strongly disagree
7. How much do you agree with this sentence: I am working at the top of my scope of practice.
   1. Strongly agree
   2. Agree
   3. Neutral
   4. Disagree
   5. Strongly agree
8. How much do you agree with this sentence: I feel supported in my job by the other professionals in clinic.
   1. Strongly agree
   2. Agree
   3. Neutral
   4. Disagree
   5. Strongly Disagree
9. How much do you agree with this sentence: The medical geneticist(s) on my team is/are open to using a new clinic model.
   1. Strongly agree
   2. Agree
   3. Neutral
   4. Disagree
   5. Strongly disagree
10. How much do you agree with this sentence: A new clinic model would allow us to see more patients per month.
    1. Strongly agree
    2. Agree
    3. Neutral
    4. Disagree
    5. Strongly disagree
11. How much do you agree with this sentence: A new clinic model would help me provide better care to my patients.
    1. Strongly agree
    2. Agree
    3. Neutral
    4. Disagree
    5. Strongly disagree
12. Do you plan on implementing a new clinic model in the future?
    1. Yes
    2. No
    3. Not sure

If yes, go to next if no it skips to after.

1. When would your clinic be most likely to start using a new clinic model?
   1. Before the end of the year
   2. Within 1 year
   3. Within 2 years
   4. Within 5 years
   5. More than 5 years
2. If a new clinic model was published from a clinic with similar patient numbers and number of professionals to yours, how likely would **you** be to want to try it out?
   1. Very likely
   2. Likely
   3. Unsure
   4. Unlikely
   5. Very unlikely
3. If a new clinic model was published from a clinic with similar patient numbers and number of professionals to yours, how likely would **your institution** be to want to try it out?
   1. Very likely
   2. Likely
   3. Unsure
   4. Unlikely
   5. Very unlikely
4. What data would be helpful in future publications related to clinic models? (free response)

**Only using non-traditional models**

1. Which of the following best describes your non-traditional clinic? (Select all that apply)

Genetic counseling only clinic

Genetic counselor working with non-genetics providers

Medical geneticist only visits

Utilization of genetic counseling assistants

Nurse practitioner working with genetic counselor in traditional model

Nurse practitioner working with medical geneticist in traditional model

Genetic counselor triage referrals

Medical geneticist goes in first, and genetic counselor always goes in

Medical geneticist sees the patient, and sees genetic counselor as needed

Genetic Counselor on-call

Genetic Counselor with general primary care provider

Genetic counselor completes phone intake

Utilization of pre-recorded videos

Utilization of chatbots

Genetic test review processes outside of clinic

Genetic counselor to non-genetics provider consult

Other (please specify)

1. Do you bill for each of these specific services?
   1. Yes
   2. No
2. Please provide any additional information pertaining to your non-traditional clinic model you believe increases access to genetic services, improves patient care, and/or team member satisfaction or dissatisfaction, please explain. (free response)
3. What is your average patient volume (unique patients for which you participated in care) per month for the non-traditional models in your clinic? This could include in-person visits, viewing patient charts, calling out results, telemedicine visits, ect.
4. In what year did your clinic implement your non-traditional clinic model?
5. What difficulties arose in implementing a new clinic model(s) at your institution?
6. Lack of administrative support
7. Logistics with setting up the new workflow
8. Budgeting
9. Referral backlog
10. Lack of confidence without medical geneticist support
11. Lack of confidence managing patient (referrals, screening, follow-up testing)
12. Interprofessional team hesitation or medical geneticist resistance
13. Difficulty delegating roles
14. Billing
15. Student involvement
16. Handling no-shows
17. Time
18. Other (free response)
19. If you were involved in implementing a non-traditional model, what advice would you provide to a colleague considering a similar model?
20. What resources were helpful in implementing non-traditional models? (put in only traditional)
    1. New features on the EMR
    2. Hiring GCA’s
    3. Clear mentoring process for new GCs
    4. Clinic meetings weekly
    5. Other (please specify)
21. What improvements have you witnessed using a non-traditional clinic model? (select all that apply)
    1. Decreased wait time
    2. Increased job satisfaction
    3. Increased in appointment flexibility
    4. Increased provider confidence in GC
    5. Increased funding for more GCs, GCAs, MDs, ect.
    6. None
    7. Unsure
    8. Other (please specify)
22. If you have practiced with both the traditional model and a non-traditional clinic model, which clinic model do you prefer between the non-traditional and traditional?
    1. Strongly prefer traditional model
    2. Prefer traditional model
    3. Neutral
    4. Prefer non-traditional model
    5. Strongly prefer non-traditional model
    6. I have only practiced with a non-traditional model.
23. How much do you agree with this sentence: I am satisfied with my job.
    1. Very satisfied
    2. Satisfied
    3. Neutral
    4. Unsatisfied
    5. Very unsatisfied
24. How much do you agree with this sentence: I am working at the top of my scope of practice.
    1. Strongly agree
    2. Agree
    3. Neutral
    4. Disagree
    5. Strongly agree
25. How much do you agree with this sentence: I feel supported in my job by the other professionals in clinic.
    1. Strongly agree
    2. Agree
    3. Neutral
    4. Disagree
    5. Strongly Disagree
    6. Strongly disagree
26. How much do you agree with this sentence: A non-traditional clinic model improves care provided to my patients.
    1. Strongly agree
    2. Agree
    3. Neutral
    4. Disagree
    5. Strongly disagree
27. How often does your clinic reassess the use of the current non-traditional clinic model?
    1. Never
    2. Monthly
    3. Annually
    4. Other (please specify)
28. If we can branch out with each selection ask, Have you made modifications to these models, if so what are they (free response)

**Combination of traditional and non-traditional model**

1. Which of the following best describes the non-traditional clinics utilized in addition to the traditional model? (Select all that apply)
   1. Genetic counseling only clinic
   2. Genetic counselor working with non-genetics providers
   3. Medical geneticist only visits
   4. Utilization of genetic counseling assistants
   5. Nurse practitioner working with genetic counselor in traditional model
   6. Nurse practitioner working with medical geneticist in traditional model
   7. Genetic counselor triage referrals
   8. Medical geneticist goes in first, and genetic counselor always goes in
   9. Medical geneticist sees the patient, and sees genetic counselor as needed
   10. Genetic Counselor on-call
   11. Genetic Counselor with general primary care provider
   12. Genetic counselor completes phone intake
   13. Utilization of pre-recorded videos
   14. Utilization of chatbots
   15. Genetic test review processes outside of clinic
   16. Genetic counselor to non-genetics provider consult
   17. Other (please specify)
2. Do you bill for each of these specific services?
   1. Yes
   2. No
3. Please provide any additional information pertaining to your non-traditional clinic model you believe increases access to genetic services, improves patient care, and/or team member satisfaction or dissatisfaction, please explain. (free response)
4. What percentage of the time do you use a non-traditional model? (slide scale)
5. In what year did your clinic implement your non-traditional clinic model?
6. What is your average patient volume (unique patients for which you participated in care with the medical geneticist) in the traditional model per month in your clinic? (Free response)
7. What is your average patient volume (unique patients for which you participated in care) per month for the non-traditional models in your clinic? This could include in-person visits, viewing patient charts, calling out results, telemedicine visits, ect. (free response)
8. What difficulties arose in implementing new clinic model(s) at your institution?
   1. Lack of administrative support
   2. Logistics with setting up the new workflow
   3. Budgeting
   4. Referral backlog
   5. Lack of confidence without medical geneticist support
   6. Lack of confidence managing patient (referrals, screening, follow-up testing)
   7. Interprofessional team hesitation or medical geneticist resistance
   8. Difficulty delegating roles
   9. Billing
   10. Student involvement
   11. Handling no-shows
   12. Time
   13. Other (free response)
9. If you were involved in implementing a non-traditional model(s), what advice would you provide to a colleague considering a similar model?
10. What improvements have you witnessed using a non-traditional clinic model? (select all that apply)
    1. Decreased wait time
    2. Increased job satisfaction
    3. Increased in appointment flexibility
    4. Increased provider confidence in GC
    5. Increased funding for more GCs, GCAs, MDs, ect.
    6. None
    7. Unsure
    8. Other (please specify)
11. How is your job different when working with the traditional clinic model versus working with the non-traditional model? (free response)
12. Which clinic model do you prefer between the non-traditional and traditional?
    1. Strongly prefer traditional model
    2. Prefer traditional model
    3. Neutral
    4. Prefer non-traditional model
    5. Strongly prefer non-traditional model
13. How much do you agree with this sentence: I am satisfied with my job. Make wording same as above add due to the utilization of a non-traditional model look at PSS and how they ask it
    1. Very satisfied
    2. Satisfied
    3. Neutral
    4. Unsatisfied
    5. Very unsatisfied
14. How much do you agree with this sentence: I am working at the top of my scope of practice.
    1. Strongly agree
    2. Agree
    3. Neutral
    4. Disagree
    5. Strongly agree
15. How much do you agree with this sentence: I feel supported in my job by the other professionals in clinic.
    1. Strongly agree
    2. Agree
    3. Neutral
    4. Disagree
    5. Strongly Disagree
    6. Strongly disagree
16. How much do you agree with this sentence: A non-traditional clinic model improves care provided to my patients
    1. Strongly agree
    2. Agree
    3. Neutral
    4. Disagree
    5. Strongly disagree
17. How often does your clinic reassess the use of the current non-traditional clinic model?
    1. Never
    2. Monthly
    3. Annually
    4. Other (please specify)

If we can branch out with each selection ask, Have you made modifications to these models, if so what are they (free response)
